# Alcohol Dehydrogenase Accentuates Ethanol-Induced Myocardial Dysfunction and Mitochondrial Damage in Mice: Role of Mitochondrial Death Pathway

**DOI:** 10.1371/journal.pone.0008757

**Published:** 2010-01-18

**Authors:** Rui Guo, Jun Ren

**Affiliations:** Center for Cardiovascular Research and Alternative Medicine, College of Health Sciences, University of Wyoming, Laramie, Wyoming, United States of America; Brigham and Women's Hospital, United States of America

## Abstract

**Objectives:**

Binge drinking and alcohol toxicity are often associated with myocardial dysfunction possibly due to accumulation of the ethanol metabolite acetaldehyde although the underlying mechanism is unknown. This study was designed to examine the impact of accelerated ethanol metabolism on myocardial contractility, mitochondrial function and apoptosis using a murine model of cardiac-specific overexpression of alcohol dehydrogenase (ADH).

**Methods:**

ADH and wild-type FVB mice were acutely challenged with ethanol (3 g/kg/d, i.p.) for 3 days. Myocardial contractility, mitochondrial damage and apoptosis (death receptor and mitochondrial pathways) were examined.

**Results:**

Ethanol led to reduced cardiac contractility, enlarged cardiomyocyte, mitochondrial damage and apoptosis, the effects of which were exaggerated by ADH transgene. In particular, ADH exacerbated mitochondrial dysfunction manifested as decreased mitochondrial membrane potential and accumulation of mitochondrial O_2_^•−^. Myocardium from ethanol-treated mice displayed enhanced Bax, Caspase-3 and decreased Bcl-2 expression, the effect of which with the exception of Caspase-3 was augmented by ADH. ADH accentuated ethanol-induced increase in the mitochondrial death domain components pro-caspase-9 and cytochrome C in the cytoplasm. Neither ethanol nor ADH affected the expression of ANP, total pro-caspase-9, cytosolic and total pro-caspase-8, TNF-α, Fas receptor, Fas L and cytosolic AIF.

**Conclusions:**

Taken together, these data suggest that enhanced acetaldehyde production through ADH overexpression following acute ethanol exposure exacerbated ethanol-induced myocardial contractile dysfunction, cardiomyocyte enlargement, mitochondrial damage and apoptosis, indicating a pivotal role of ADH in ethanol-induced cardiac dysfunction possibly through mitochondrial death pathway of apoptosis.

## Introduction

Alcohol (ethanol) exposure often results in the development of alcoholic cardiomyopathy characterized by cardiomegaly (dilated cardiomyopathy), disruption of myofibrillary architecture and myocardial dysfunction [1], [2]. Although a number of scenarios have been speculated towards the onset and progression of ethanol-induced myopathic changes including direct cardiotoxicity of ethanol and its metabolites [3], oxidative stress and accumulation of fatty acid ethyl esters [4], the ultimate culprit factor(s) behind alcohol-elicited cardiac damage remains elusive. Acetaldehyde, the primary metabolic product of ethanol, has drawn some recent attentions as a candidate toxin for the onset and development of alcoholic cardiomyopathy [5]. Data from our laboratory have shown that acetaldehyde directly compromises myocardial excitation-contraction coupling, sarco(endo)plasmic reticulum (SR) Ca^2+^ release and cardiac contractile function [5]–[8]. Meanwhile, facilitated clearance of acetaldehyde via mitochondrial aldehyde dehydrogenase (ALDH-2) was shown to be beneficial in alleviating acute and chronic ethanol exposure-induced contractile dysfunction and/or myocardial hypertrophy [9], [10], further supporting the detrimental role of acetaldehyde in alcohol-induced myocardial damage. Nonetheless, the precise mechanism of action behind the acetaldehyde-induced unfavorable myocardial functional and morphological changes following either acute or chronic ethanol exposure remains elusive. Given that apoptosis and mitochondrial damage are commonly present in response to ethanol challenge and are thought to play an essential role in alcoholism-elicited organ damage and complications [9], [11], our current study was designed to address the role of mitochondrial function and apoptosis in ethanol-induced myocardial dysfunction. Here we took advantage of the novel transgenic mouse model generated in our labs with the cardiac-specific overexpression of alcohol dehydrogenase (ADH), which mimics an “acetaldehyde overloaded” model of alcoholic cardiomyopathy [12]. Myocardial mitochondrial damage was assessed using mitochondrial superoxide (O_2_^•−^) accumulation and mitochondrial membrane potential. Mitochondria are known to play a key role in the maintenance of cardiac function and morphology through regulation of reactive oxygen species production and apoptosis [11]. Mitochondria are often themselves targets of oxidative stress and contribute to mechanisms by which oxidative stress-related cell signals control cardiac contractile function [11], [13]. We further examined the roles of the two main apoptotic domains including one through activated death receptors in the cell surface (extrinsic pathway) and another via signals originated within the cell involving mitochondria as either an initiator or a magnifier (intrinsic pathway) [14]. The death receptor pathway is usually triggered by the linkage of specific ligands to membrane receptors including tumor necrosis factor α (TNF-α) and Fas receptor [14]. To this end, expression of TNF-α, Fas, Fas ligand (FasL), Caspase-8 and pro-caspase-8 was examined in wild-type FVB and ADH hearts following acute ethanol challenge. To monitor the change in mitochondrial death domain, cytosolic accumulation of pro-caspase-9, cytochrome C and apoptosis inducing factor (AIF) was examined. TUNEL assay and levels of the pro-apoptotic proteins Bax and Caspase-3 as well as the anti-apoptotic protein Bcl-2 were used as for overall assessment of apoptosis.

## Materials and Methods

### Experimental Animals and Acute Ethanol Exposure

All animal procedures were conducted in accordance with humane animal care standards outlined in the NIH Guide for the Care and Use of Experimental and were approved the University of Wyoming Animal Care and Use Committee. Production of the ADH transgenic mice was described in detail previously [15]. In brief, using the albino Friend Virus-B type (FVB) mice, the cDNA for murine class I ADH was inserted behind mouse α-myosin heavy chain promoter to achieve cardiac-specific overexpression. This cDNA was chosen because class I ADH is the most efficient in the oxidation of ethanol. A second transgene with a cDNA encoding tyrosinase was co-injected with ADH. This enzyme produces coat color pigmentation in albino mice and was used to conveniently identify transgenic animals. All mice were housed in a temperature-controlled room under a 12 hr/12 hr-light/dark and allowed access to tap water *ad libitum*. For acute ethanol challenge, adult male FVB and ADH mice (4–6 month-old) were injected intraperitoneally with ethanol (3 g/kg/d) for 3 consecutive days prior to euthanasia under anesthesia (ketamine/xylazine: 3∶1, 1.32 mg/kg, i.p.).

### Assessment of Ethanol and Acetaldehyde Levels

Upon sacrifice under anesthesia, blood plasma was collected and was stored in sealed vials at −80°C. Immediately before analysis, the samples were warmed to 25°C. A 2 ml aliquot of the headspace gas from each vial was removed through the septum on the cap with a gas tight syringe and transferred to a 200 µl loop injection system on a Hewlett-Packard 5890 gas chromatograph (GC) equipped with a flame ionization detector. Ethanol, acetaldehyde and other components were separated on a 9-meter VOCOL capillary column (Supelco) with film with 1.8 µm thickness and an inner diameter of 0.32 mm. The temperature was held isothermally at 30°C, and the carrier gas was helium at a flow rate of 1.8 ml/min. Under these conditions, separation of acetaldehyde from ethanol and other compounds was complete in one minute. Quantitation was achieved by calibrating the GC peak areas against those from headspace samples of known ethanol and acetaldehyde standards, over a similar concentration range as the tissue samples in the same buffer [16].

### Mouse Heart Perfusion

Isolated mouse hearts were retrogradely perfused with a Krebs-Henseleit buffer containing 7 mM glucose, 0.4 mM oleate, 1% BSA and a low fasting concentration of insulin (10 µU/ml). Hearts were perfused at a constant flow of 4 ml/min (equal to an aortic pressure of 80 cmH_2_O) at baseline for 60 min. A fluid-filled latex balloon connected to a solid-state pressure transducer was inserted into the left ventricle through a left atriotomy to measure pressure. LVDP, the first derivative of LVDP (± dP/dt) and heart rate were recorded using a digital acquisition system at a balloon volume which resulted in a baseline LV end-diastolic pressure of 5 mmHg [17].

### Histological Examination

Following anesthesia, hearts were excised and immediately placed in 10% neutral-buffered formalin at room temperature for 24 hrs after a brief rinse with PBS. The specimen were embedded in paraffin, cut in 5 µm sections and stained with hematoxylin and eosin (H&E). Cardiomyocyte cross-sectional areas were calculated on a digital microscope (x400) using the Image J (version1.34S) software [9].

### Isolation of Murine Cardiomyocytes

After ketamine/xylazine sedation, hearts were removed and perfused with Krebs-Henseleit bicarbonate (KHB) buffer containing (in mM): 118 NaCl, 4.7 KCl, 1.2 MgSO_4_, 1.2 KH_2_PO_4_, 25 NaHCO_3_, 10 HEPES and 11.1 glucose. Hearts were digested with collagenase D for 20 min. Left ventricles were removed and minced before being filtered. Myocyte yield was 50%–70% which was not overtly affected by ADH or ethanol challenge [9].

### MitoSOX Red Fluorescence Measurement of Mitochondrial O_2_^•−^

Cardiomyocytes from FVB and ADH mice with or without ethanol treatment were loaded with MitoSOX Red (2 µM, Molecular Probes) for 10 min. Maximum fluorescence uptake interval was evaluated by preliminary experiments. After 1 hr's incubation at 37°C, cells were rinsed with the perfusion buffer and MitoSOX Red fluorescence intensity was captured at 510/580 nm using an Olympus BX51 microscope equipped with a digital cooled charged-coupled device camera. InSpeck microspheres (Molecular Probes) were used to calibrate MitoSOX Red fluorescence by calculating the ratio of myocyte fluorescent intensities to the fluorescent beads [18]. To assess the effect of the ethanol metabolite acetaldehyde on mitochondrial O_2_^•−^ generation, freshly isolated cardiomyocytes from non-ethanol-treated FVB mice were incubated with acetaldehyde (100 µM) for 4 hrs at 37°C prior to determination of the MitoSOX Red fluorescence.

### Measurement of Mitochondrial Membrane Potential

Mitochondrial membrane potential (ΔΨ_m_) was detected in cardiomyocytes suspended in a HEPES-saline buffer [19]. Briefly, after a 10-min incubation with 5 µM JC-1 at 37°C, cells were washed twice by sedimentation using HS buffer free of JC-1. Cardiomyocytes were examined every 10 min for 120 min under a confocal laser scanning microscope (Leica TCS SP2) at excitation wavelength of 490 nm and the emission fluorescence was recorded at 530 nm (monomer form of JC-1, green) and 590 nm (aggregate form of JC-1, red). Results in fluorescence intensity were expressed as 590-to-530-nm emission ratio. The mitochondrial uncoupler carbonyl cyanide m-chlorophenylhydrazone (CCCP, 10 µM) was used as a positive control for mitochondrial membrane potential measurement [20].

### TUNEL Staining

TUNEL (terminal deoxynucleotidyl transferase-mediated dUTP nick-end labeling) assessment of myonuclei positive for DNA strand breaks was determined using a fluorescence detection kit (Roche Applied Science, Indianapolis, IN) and fluorescence microscopy. After perfusion, mouse hearts were removed and fixed in 4% paraformaldehyde overnight at room temperature. Cross sections (5 µm) were placed in a cryostat (−23°C) and fixed in 4% paraformaldehyde 20 min and then fixed Sections were permeabilized with 0.1% Triton X-100 in 0.1% sodium citrate for 2 min on ice. TUNEL reaction mixture containing terminal deoxynucleotidyl transferase (TdT), fluorescein-dUTP was added to the sections in 50-µl drops and incubated for 60 min at 37°C in a humidified chamber in the dark. The sections were rinsed three times in PBS for 5 min each. Following embedding, sections were visualized with an Olympus BX-51 microscope equipped with an Olympus MaguaFire SP digital camera. DNase I and label solution were used as positive and negative controls. To determine the percentage of apoptotic cells, the TUNEL-positive nuclei and TUNEL-negative cells were counted using the ImagePro image analysis software (Media Cybernetics, Bethesda, MD) [21].

### Western Blot Analysis

The total and cytosolic fractions of protein were prepared as described [9], [22]. Samples containing equal amount of proteins were separated on 10% SDS-polyacrylamide gels in a minigel apparatus (Mini-PROTEAN II, Bio-Rad) and transferred to nitrocellulose membranes. The membranes were blocked with 5% milk in TBS-T, and were incubated overnight at 4°C with anti-ANP, anti-Bax, anti-Bcl-2, anti-caspase-3, anti-pro-caspase-8, anti-pro-caspase-9, anti-TNFα, anti-Fas receptor, anti-Fas L, anti-AIF, and anti-cytochrome C antibodies. After washing blots to remove excessive primary antibody binding, blots were incubated for 1 hr with horseradish peroxidase (HRP)–conjugated secondary antibody (1∶5,000). Antibody binding was detected using enhanced chemiluminescence (Amersham Pharmacia, Piscataway, NJ), and film was scanned and the intensity of immunoblot bands was detected with a Bio-Rad Calibrated Densitometer (Model: GS-800). All tissue samples were run in duplicates. α-Tubulin was used as the loading control.

### Data Analysis

Data are Mean ± SEM. Difference was calculated by repeated measures analysis of variance (ANOVA) followed by a Tukey's *post hoc* analysis. A p value<0.05 was considered significant.

## Results

### General Features and Whole Heart Function of FVB and ADH Mice Treated with Alcohol

**Figure 1:**
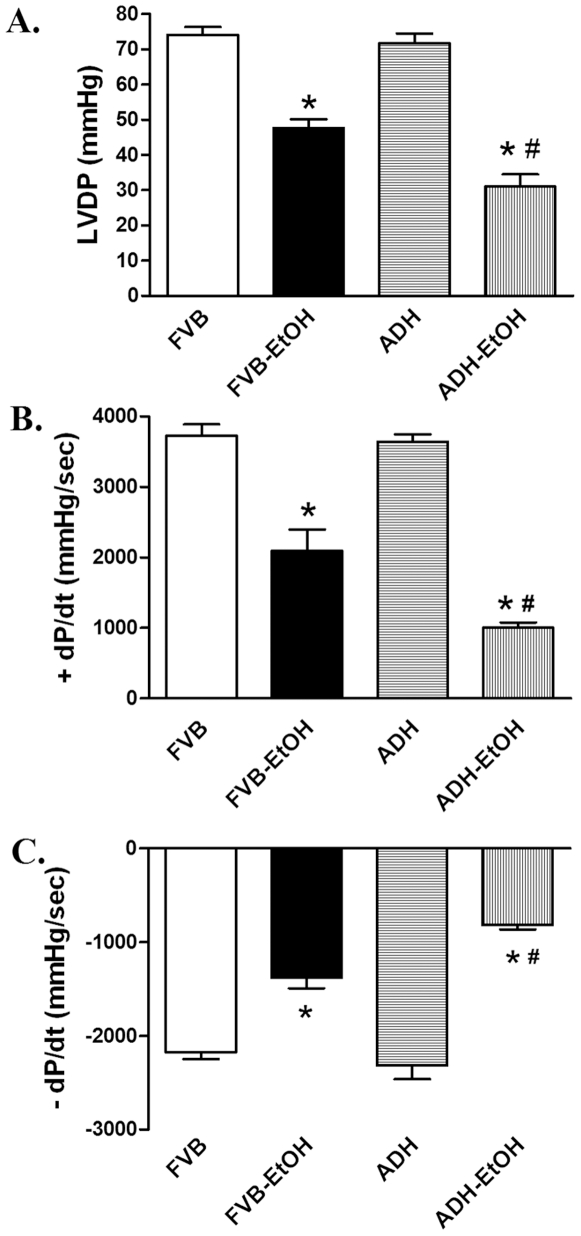
Effect of ethanol exposure on cardiac function: Effect of acute ethanol exposure on cardiac contractile function using a Langendorff perfusion system in FVB and ADH mice. A: Left ventricular developing pressure (LVDP); B and C: Maximal velocity of pressure development (+dP/dt) and decline (−dP/dt). Mean ± SEM, n = 5–10 hearts per group, * p<0.05 *vs.* FVB, # p<0.05 *vs.* FVB-EtOH group.

Neither ethanol treatment nor ADH transgene altered body and organ weights or organ size (shown as the organ-to-body weight ratio). As expected, acute ethanol exposure elicited comparable elevations in blood alcohol level, which was minimal in the non-ethanol-treated mice. Cardiac acetaldehyde levels were significantly increased following ethanol challenge, the effect of which was exacerbated by the ADH transgene (Table 1). Assessment of whole heart function including LVDP, maximal velocity of pressure development and decline (± dP/dt) revealed a significant decline following ethanol treatment, the effect of which was accentuated by ADH (Fig. 1).

**Table 1:** Biometric parameters of FVB and ADH mice challenged with ethanol.

Parameter	FVB	FVB-EtOH	ADH	ADH-EtOH
Body Weight (g)	26.8±0.9	26.2±0.8	28.3±0.9	28.6±0.9
Heart Weight (mg)	140±6	130±7	140±5	141±6
Heart/Body Weight (mg/g)	5.23±0.16	4.94±0.19	5.69±0.18	4.95±0.23
Liver Weight (g)	1.26±0.08	1.30±0.05	1.40±0.05	1.39±0.04
Liver/Body Weight (mg/g)	47.2±2.5	49.4±0.8	49.7±2.1	49.3±1.8
Kidney Weight (g)	0.31±0.02	0.33±0.02	0.34±0.02	0.37±0.04
Kidney/Body Weight (mg/g)	11.5±0.3	12.6±0.4	12.1±0.6	13.2±0.5
Blood Alcohol (mg/dl)	Undetectable	59.3±10.3*	Undetectable	62.7±21.5*
Cardiac Acetaldehyde Levels (nmol/mg)	0.87±0.53	35.3±4.2*	0.60±0.40	99.9±12.0*,^#^

Mean ± SEM, n = 12–15 mice per group, undetectable: <2.5 mg/dl, *p<0.05 *vs.* FVB group, ^#^p<0.05 vs. FVB-EtOH group.

### Effect of Acute Ethanol Exposure on Myocardial Histology in FVB and ADH Mice

**Figure 2:**
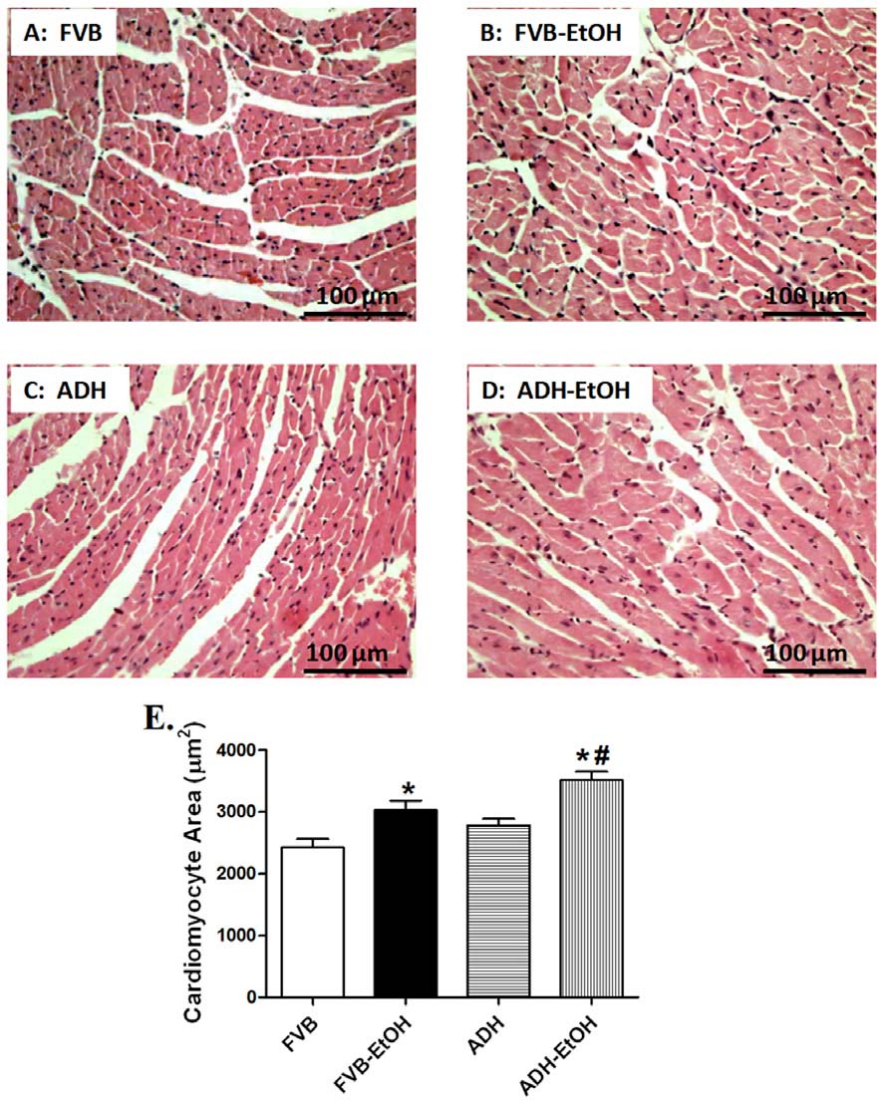
Histological analyses following ethanol exposure: Histological analyses hearts from FVB and ADH mice with or without ethanol exposure. A - D: H&E staining micrographs of transverse sections of left ventricular myocardium (x 400) from FVB, FVB-EtOH, ADH and ADH-EtOH groups; E: Quantitative analysis of cardiomyocyte cross-sectional (transverse) area using measurements of ∼150 cardiomyocytes from 3–5 mice per group. Mean ± SEM, * p<0.05 *vs.* FVB, # p<0.05 *vs.* FVB-EtOH group.

To assess the impact ADH on myocardial histology following acute ethanol challenge, the cardiomyocyte cross-sectional area was examined. In the H&E staining sections, acute ethanol challenge increased the cardiomyocyte transverse cross-section area. The ethanol-induced increase in cardiomyocyte cross-sectional area was significantly augmented by the ADH transgene (Fig. 2).

### Effect of Acute Ethanol Exposure on Mitochondrial O_2_^•−^ Production and Membrane Potential

**Figure 3:**
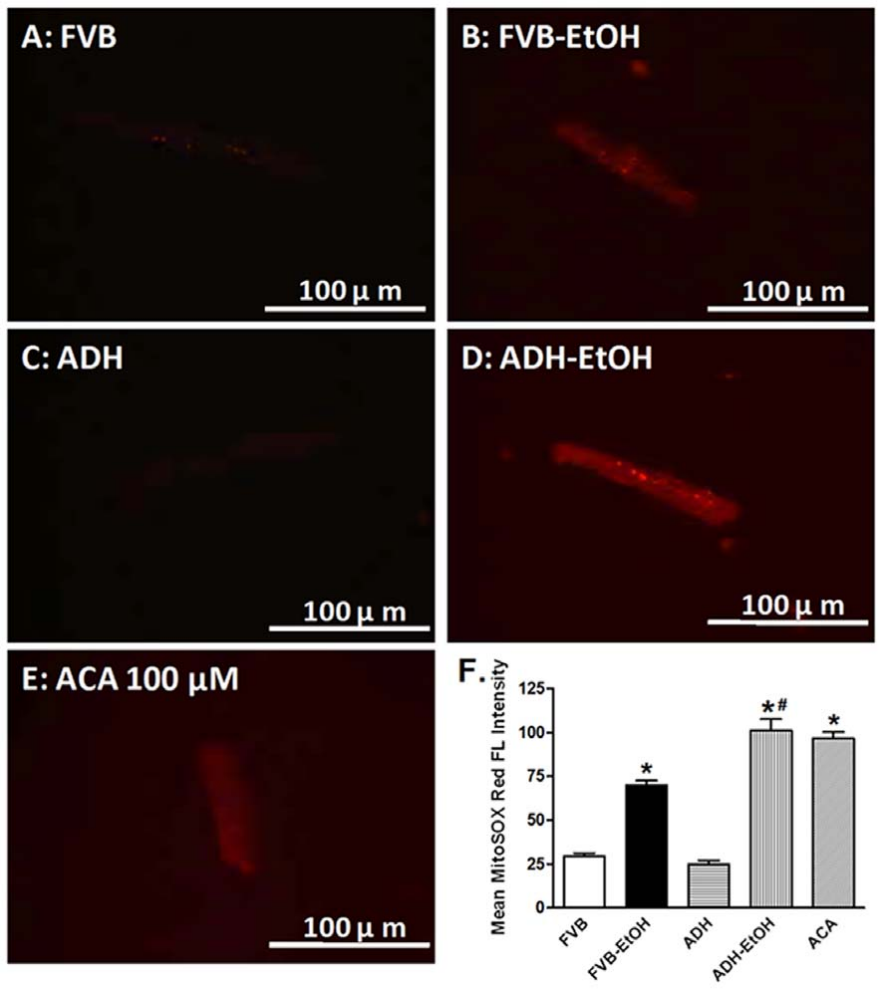
Mitochondrial O_2_^•−^ generation following ethanol exposure: Mitochondrial O_2_^•−^ generation using the MitoSOX Red probe in cardiomyocytes from FVB and ADH mice with or without acute ethanol exposure. Cohorts of non-ethanol-treated FVB cardiomyocytes were incubated with the ADH enzymatic metabolite of ethanol, acetaldehyde (ACA, 100 µM), for 4 hrs at 37°C prior to MitoSOX Red measurement. A–E: Representative fluorescence images (40x) from FVB, FVB-EtOH, ADH, ADH-EtOH and FVB-ACA groups. F: Pooled data. Mean ± SEM, n = 15–20 fields per group, * p<0.05 *vs.* FVB, # p<0.05 *vs.* FVB-EtOH group.

**Figure 4:**
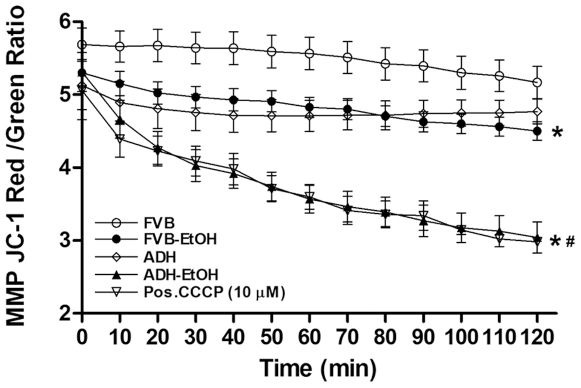
Mitochondrial function following ethanol exposure: Cardiomyocyte mitochondrial membrane potential (MMP) in FVB and ADH mice with or without acute ethanol exposure. JC-1 fluorochrome was shown as the ratio of red to green fluorescence. CCCP was used a positive control. Mean ± SEM, n = 9–14 cells per group, * p<0.05 *vs.* FVB, # p<0.05 *vs.* FVB-EtOH group.

To evaluate mitochondrial integrity and function, mitochondrial O_2_^•−^ production and membrane potential were detected using the MitoSOX Red and JC-1 fluorescent probes, respectively. Our data revealed that acute ethanol exposure significantly promoted mitochondrial O_2_^•−^ production and decreased mitochondrial membrane potential in cardiomyocytes, the effects of which were accentuated by ADH. The ADH transgene itself did not elicit any significant effect on mitochondrial O_2_^•−^ production or mitochondrial membrane potential (Fig. 3 and Fig. 4). Moreover, the ADH enzymatic metabolite of ethanol, acetaldehyde (100 µM), significantly promoted mitochondrial O_2_^•−^ production (Fig. 3F).

### Effect of Acute Ethanol Exposure on Myocardial Apoptosis in FVB and ADH Mice

**Figure 5:**
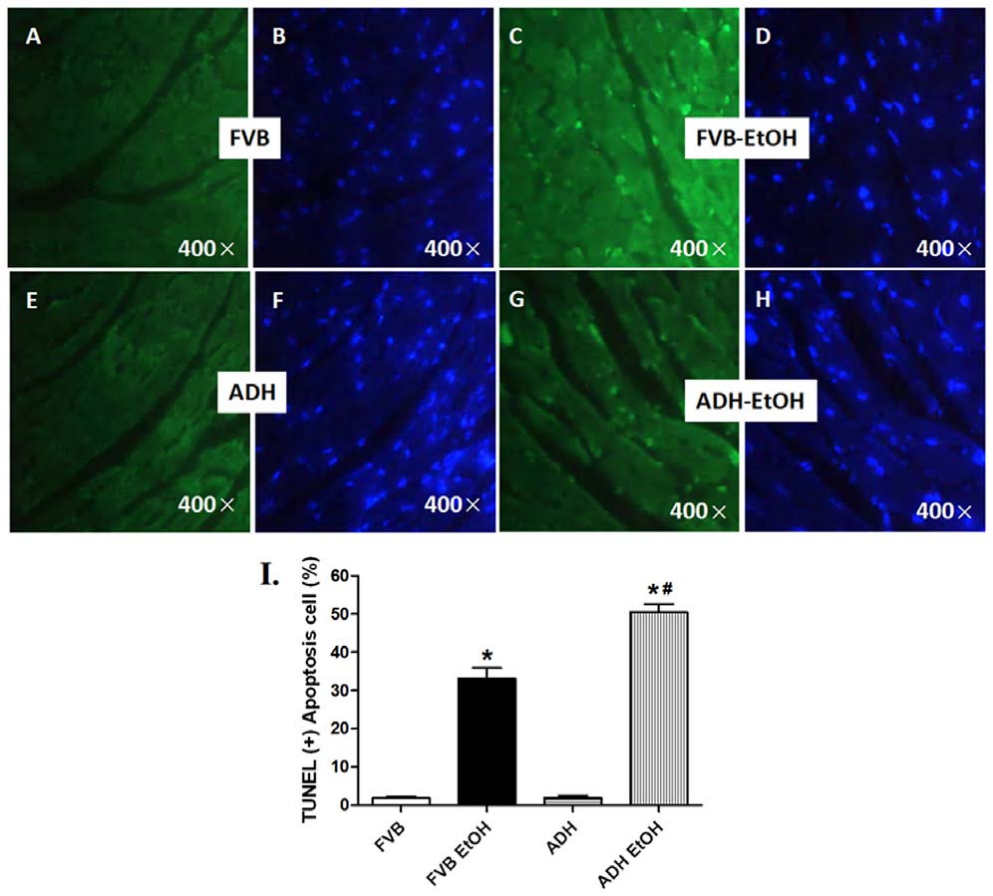
TUNEL staining following ethanol exposure: Photomicrograph showing TUNEL staining in cardiomyocytes from FVB and ADH mice with or without acute ethanol treatment. TUNEL positive nuclei were visualized with fluorescein (green). A: FVB; C: FVB-EtOH; E: ADH; and G: ADH-EtOH. All nuclei were stained with DAPI shown in blue color. B: FVB; D: FVB-EtOH; F: ADH; and H: ADH-EtOH. Pooled data are shown in panel I. Mean ± SEM, n = 4–6 mice per group, * p<0.05 *vs.* FVB, # p<0.05 *vs*. FVB-EtOH group.

Acute ethanol exposure is often associated with enhanced apoptosis [9]. The acute ethanol challenge-elicited effect on myocardial mitochondrial damage was further supported by apoptotic assay using TUNEL staining. The TUNEL-positive nuclei visualized in fluorescein green as a percentage of all nuclei stained with DAPI (blue) was significantly higher in myocardium from acute ethanol-treated FVB mice, the effect of which was exacerbated by ADH. ADH transgene itself did not affect the TUNEL-positive nuclei in the absence of ethanol exposure (Fig. 5).

### Effect of Acute Ethanol Challenge on ANP and Apoptotic Protein Makers in FVB and ADH Mice

**Figure 6:**
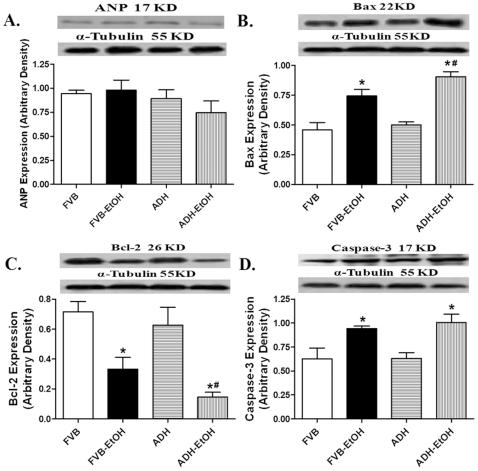
Cardiac hypertrophy and apoptosis following ethanol exposure: Expression of the hypertrophic marker ANP and apoptosis-related proteins in myocardium from FVB and ADH mice with or without acute ethanol exposure. A: ANP; B: Bax; C: Bcl-2; and D: Caspase-3. Insets: Representative gel blots depicting expression of ANP, Bax, Bcl-2, Caspase-3 and α-Tubulin (loading control). Mean ± SEM, n = 5–10 samples per group, all samples were in duplicates with the average being used, * p<0.05 *vs.* FVB, # p<0.05 *vs*. FVB-EtOH group.

**Figure 7:**
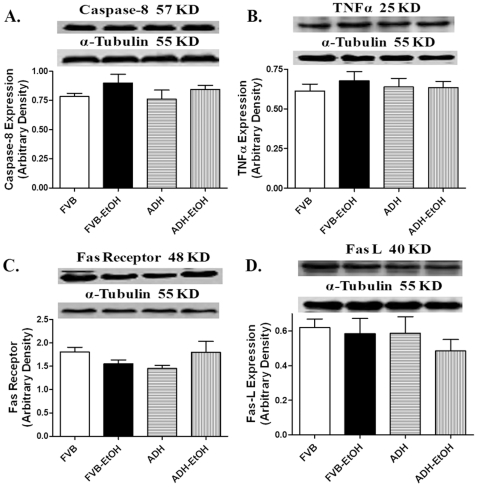
Death receptor pathway in myocardium following ethanol exposure: Expression of Caspase-8 (A), TNFα (B), Fas receptor (C) and Fas Ligand (Fas L, D) in myocardium from FVB and ADH mice with or without acute ethanol exposure. Insets: Representative gels using specific antibodies. Mean ± SEM, n = 3–6 samples per group, all samples were in duplicates with the average being used.

**Figure 8:**
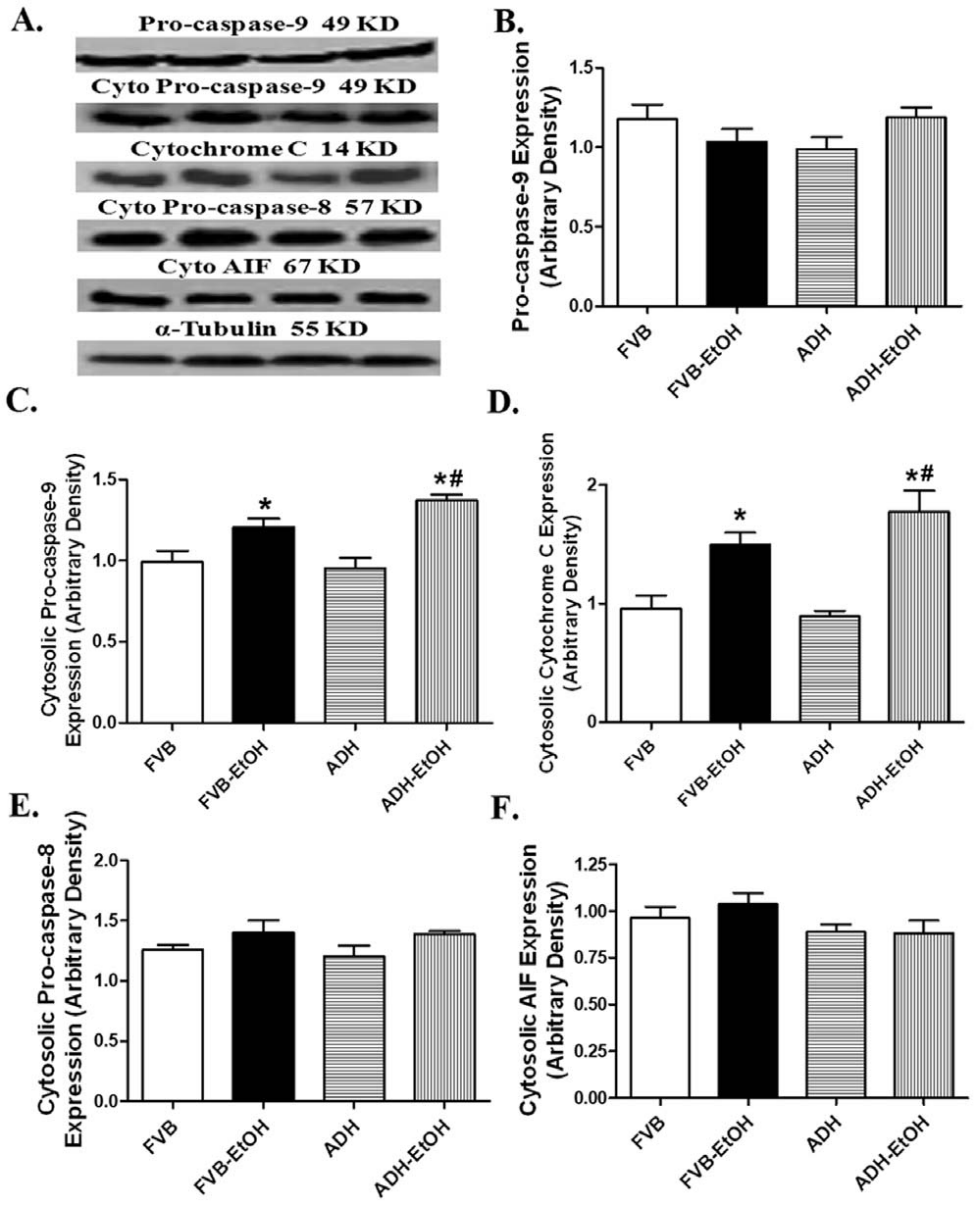
Mitochondrial death pathway in myocardium following ethanol exposure: Expression of pro-caspase-9 (B), cytosolic pro-caspase-9 (C), cytosolic cytochrome C (D), cytosolic pro-caspase-8 (E) and cytosolic AIF (F) in myocardium from FVB and ADH mice with or without acute ethanol exposure. Panel A depicts representative gels using specific antibodies. Mean ± SEM, n = 4–8 samples per group, all samples were in duplicates with the average being used, * p<0.05 *vs.* FVB group.

Our data revealed that neither acute ethanol challenge nor ADH transgene affected the hypertrophic marker ANP expression. In line with the TUNEL assay observation, our further results indicated that ethanol treatment significantly increased expression of the pro-apoptotic proteins Bax and Caspase-3 while decreasing the level of the anti-apoptotic protein Bcl-2. Although ADH failed to alter the ethanol-induced response of Caspase-3, it significantly augmented ethanol-elicited responses in Bax and Bcl-2. ADH transgene itself did not elicit any effect on the expression of Bax, Bcl-2 and Caspase-3 in the absence of ethanol treatment (Fig. 6). We went on to examine the involvement of the death receptor and mitochondrial death pathways in ADH and ethanol-associated apoptotic effects. Neither ethanol nor ADH transgene affected the levels of the main death receptor apoptotic proteins TNFα, Fas receptor and Fas ligand (FasL) and Caspase-8 (Fig. 7). Examination of the mitochondrial death pathway depicted that acute ethanol treatment significantly promoted cytosolic accumulation of cytochrome C and pro-caspase-9, the effect of which was augmented by ADH. Neither ethanol nor ADH altered the levels of total pro-caspase-9, cytosolic AIF and the death receptor mediator cytosolic pro-caspase-8 (Fig. 8).

## Discussion

The hallmarks of alcoholic cardiomyopathy include compromised cardiac morphology and myocardial contractility [1], [2], [5]. This is coincided with our observations of reduced myocardial contraction and enlarged cardiomyocyte area in ethanol-challenged murine hearts. Furthermore, data from our present study revealed overt mitochondrial damage and apoptosis following ethanol challenge, which may contribute to the ethanol-elicited myocardial histological and functional alterations. More strikingly, our study provided evidence for the first time that cardiac mitochondrial damage and apoptosis following ethanol exposure may be exacerbated by overexpression of ADH, which produces much more local acetaldehyde in the hearts consistent with the previous findings [15], [16]. These observations are in favor of the notion that facilitated ethanol metabolism via ADH enzyme exacerbates ethanol-induced myocardial dysfunction, histological alteration and apoptosis possibly related to mitochondrial damage.

Data from our present study revealed that ADH accentuated ethanol-induced cardiomyocyte hypertrophy with little change in gross weight of the heart or expression of the hypertrophic marker ANP. These seemingly conflicting data may be a concerted result from hypertrophy and apoptosis in murine cardiomyocytes. An earlier study using the same ADH transgenic mice noted overt cardiac hypertrophy and upregulated expression of the hypertrophic markers α-skeletal actin and atrial natriuretic factor in ADH but not FVB mice after 10 weeks of alcohol feeding [23]. To this end, it is not surprising for the lack of change in ANP in response to acute ethanol challenge in our experimental settings. The enlarged cardiomyocyte size may be a result of certain hemodynamic changes following acute ethanol challenge or binge drinking although further study is warranted. Acetaldehyde is known to trigger both oxidative stress and apoptosis via activation of stress signaling such as c-Jun phosphorylation [5], [24], [25]. This is supported by our experimental findings of elevated O_2_^•−^ production and TUNEL-positive apoptotic cells in ADH murine hearts following ethanol challenge. Enhanced O_2_^•−^ and oxidative stress are known to induce hypertrophy in cardiomyocytes. As the major metabolite of ethanol, acetaldehyde enhances free radical generation through aldehyde oxidase and xanthine oxidase-associated oxidation, leading to accumulation of O_2_^•−^ as shown in our present study [5], [26]–[28]. Our earlier report indicated that ADH produced greater levels of lipid peroxidation and protein carbonyls in hearts from the alcohol-fed mice [16], indicating a key role of free radical formation in alcohol- and acetaldehyde-induced cardiac damage. To the contrary, the occurrence of apoptosis contributes to the loss of cardiomyocytes, which is deemed as a predictor of adverse outcomes for cardiac diseases and eventually heart failure [29]. With the concurrent hypertrophy and loss of cell number in cardiomyocytes, it is not surprising to find the unchanged gross heart weight in response to acute ethanol exposure.

Mitochondrial integrity plays a pivotal role for cell survival and function [11]. Loss of mitochondrial integrity leads to the development of several diseases such as neurodegenerative disorders, diabetes and ischemia reperfusion-induced heart damage. Recently, mitochondrial dysfunction also received some attentions in the onset of alcoholic complications [11]. Data from our current study revealed elevated mitochondrial O_2_^•−^ production and reduced mitochondrial membrane potential in hearts following acute ethanol exposure. More intriguingly, the ethanol-induced changes in mitochondrial membrane potential and mitochondrial O_2_^•−^ production were exaggerated by the ADH transgene (or mimicked by the ethanol metabolite acetaldehyde). These observations were in line with the changes in myocardial contractility and histology in both FVB and ADH mice following ethanol exposure, suggesting the essential role of mitochondria in ADH-induced exacerbation of myocardial injury in response to ethanol exposure.

Mitochondrial damage has been demonstrated to result in apoptosis through mitochondrial pathways [11]. Apoptosis or programmed cell death plays a key role in the pathogenesis of a variety of diseases including atherosclerosis, myocardial ischemia and reperfusion injury, diabetic cardiomyopathy and alcoholic cardiomyopathy [30]–[33]. Apoptosis is closely associated with a number of cardiovascular anomalies such as myocardial infarction, dilated cardiomyopathy, end-stage heart failure, ventricular dysplasia, hypertrophic cardiomyopathy and ischemia reperfusion injury in both patients and animal models [34]–[39]. As mentioned earlier, two major defined pathways are involved in the apoptotic signaling cascade in the heart namely the death receptor and mitochondria pathways, both of which mainly depend on the activation of caspase [40], [41].

In this study, the decreased expression of Bcl-2, up-regulated expression of caspase-3 and Bax, as well as increased TUNEL positive cells depicted the presence of a global myocardial apoptosis following acute ethanol exposure. Our observation of significantly increased cytosolic expression of cytochrome C and pro-caspase-9 in ethanol-treated mice depicts an essential role of the mitochondrial death pathway in ethanol-induced apoptosis. The fact that the ADH transgene augmented the ethanol-induced cytosolic accumulation of pro-caspase-9 and cytochrome C further substantiated the critical role of acetaldehyde in ethanol-induced mitochondrial damage, which is consistent with the ADH-accentuated mitochondrial O_2_^•−^ production and mitochondrial membrane potential loss following acute ethanol challenge. It has been demonstrated that pro-caspase-8 and pro-caspase-9 are predominantly localized in mitochondria which are released into cytoplasm upon permeabilization of the outer mitochondrial membrane upon apoptosis stimulation or oxidative stress [42], [43]. AIF, another important factor normally localizes to the mitochondrial inter-membrane space, plays a critical role in the caspase-independent apoptosis [44]. Our finding of unaltered cytosolic AIF expression does not seem to favor the involvement of a caspase-independent apoptotic process in ethanol-induced cell death. It should be mentioned that our results cannot directly address the intimate interplay between mitochondrial damage and myocardial dysfunction in these mice following acute ethanol exposure. Our current findings of unchanged expression of TNF-α, Fas receptor, Fas L, caspase-8 and pro-caspase-8 in FVB and ADH mice in response to ethanol exposure suggest a minimal role of the death receptor pathway in ethanol-and ADH-elicited apoptotic responses.

In summary, the present study has provided convincing evidence that cardiac overexpression of ADH exacerbated acute ethanol exposure-induced myocardial contractile dysfunction associated with mitochondrial damage and apoptosis, supporting an essential role of acetaldehyde and mitochondrial dysfunction in ethanol-elicit alcoholic myopathic alteration. Although it is still premature to discern the precise contributions from various death pathways including the newly identified autophagy mechanism to ethanol-induced cell death and tissue injury, our data should shed some lights towards a better understanding of the role of mitochondria and mitochondrial death pathway in alcohol-induced myocardial dysfunction. Although certain mitochondria- centered medicinal product (e.g., MacroMicro™ Cleanse & Detox) has been developed to improve mitochondrial function against acetaldehyde-induced handover and other complications following alcohol intake, further scrutiny is required to unveil the clinical value of mitochondrial protection under the state of both binge drinking and chronic alcoholism.
